# Changing Trend of Neonatal Septicemia and Antibiotic Susceptibility Pattern of Isolates in Nepal

**DOI:** 10.1155/2019/3784529

**Published:** 2019-02-06

**Authors:** Sangita Thapa, Lokendra Bahadur Sapkota

**Affiliations:** ^1^Department of Clinical Microbiology and Immunology, Chitwan Medical College Teaching Hospital, 44200 Chitwan, Nepal; ^2^Department of Biochemistry, Chitwan Medical College Teaching Hospital, 44200 Chitwan, Nepal

## Abstract

**Background:**

Neonatal septicemia is one of the most common leading reasons for neonatal morbidity and mortality in developing countries. Frequent monitoring on pathogens with recent updates and their antimicrobial sensitivity pattern is mandatory for the better treatment. The aim of the study was to determine the bacteriological profile of neonatal septicemia and their antibiotic susceptibility pattern.

**Methods:**

This was a cross-sectional study conducted in Outpatient Department (OPD), Neonatal Intensive Care Unit (NICU), and Pediatrics Ward of Chitwan Medical College Teaching Hospital (CMCTH), Bharatpur, Nepal. Blood cultures were performed on all suspected neonates attending to the hospital with a clinical analysis of neonatal septicemia. Isolated organism was identified by the standard microbiological protocol and antibiotic sensitivity testing was done by Kirby-Bauer disk diffusion method.

**Results:**

Out of 516 specimens, bacterial growth was obtained in 56 specimens (10.8%). Prevalence of early onset sepsis was higher 35 (62.5%) in neonates compared to late onset sepsis 21 (37.5%). Majority of neonatal septicemia were caused by gram-negative isolates 39 (69.6%).* Acinetobacter *species 18 (32.1%) was most commonly isolated organism followed by* Staphylococcus aureus *11 (19.6%). The predominant isolate in early onset septicemia was* Acinetobacter *species 18 (32.1%) and* Staphylococcus aureus *9 (16%) and in late onset septicemia was* Staphylococcus aureus *11 (19.6%) and* Acinetobacter *species 5 (8.9%).* Staphylococcus aureus *and coagulase-negative* Staphylococci *displayed highest susceptibility towards vancomycin, amikacin, teicoplanin, and meropenem. Gram-negative isolates showed susceptibility towards amikacin, piperacillin/tazobactam, meropenem, ofloxacin, and gentamicin.

**Conclusions:**

* Acinetobacter *species and* Staphylococcus aureus *remain the most predominant organisms responsible for neonatal septicemia in a tertiary care setting and demonstrate a high resistance to the commonly used antibiotics. Above all, since the rate of* Acinetobacter *species causing sepsis is distressing, inspiring interest to control the excess burden of* Acinetobacter *species infection is mandatory.

## 1. Background

Neonatal septicemia is a clinical condition characterized by systemic signs and symptoms due to bacteremia in the first month of the life. Neonatal septicemia is considered one of the leading causes of neonatal mortality globally, more in developing countries like Nepal [1]. According to World Health Organization (WHO), every year an estimated 1.6 million neonatal deaths occur globally with 40% of all neonatal deaths occurring in developing countries [2]. According to Nepal Demographic and Health Survey 2016, national neonatal mortality rate was 21/1000 live births. Infections including sepsis contributed to 16% of the neonatal mortality [3].

The risk factors those may be associated with neonatal septicemia are premature rupture of membrane, prolonged rupture, prematurity, urinary tract infection, poor maternal nutrition, low birth weight, birth asphyxia, and congenital anomalies [4]. The spectrum of organisms causing neonatal septicemia shows variation in different countries and even varies in hospitals of the same region. Moreover, group of organisms may be replaced by others over a period of time. In developed countries, gram-negative organisms are the most common organisms of neonatal septicemia [5].

Neonatal septicemia may be divided into two types. The infection acquired within 72 hrs of age is known as early onset neonatal septicemia and the common bacteria associated with it are group B* Streptococcus*,* Escherichia coli *(*E. coli*), coagulase-negative* Staphylococci *(CONS),* Haemophilus influenzae *(*H. influenzae*), and* Listeria monocytogenes*. Similarly, the infection acquired after 72 hours of age is known as late onset neonatal septicemia and the most common causative agents are CONS,* Staphylococcus aureus *(*S. aureus*),* Klebsiella pneumonia*e (*K. pneumoniae*),* E. coli*,* Pseudomonas aeruginosa *(*P. aeruginosa*), and* Acinetobacter *spp. [6].

Neonatal septicemia can be life threatening if proper treatment is not given in time. Neonatal septicemia is difficult to diagnose clinically as it presents with nonspecific signs and symptoms. Though various diagnostic modalities exist for neonatal septicemia including C-reactive protein, complete blood count, platelet count, and erythrocyte sedimentation rate, yet blood culture is the gold standard [7].

Knowledge regarding common pathogens and antimicrobial susceptibility pattern causing neonatal septicemia is essential in order to select appropriate antibiotic therapy to decrease neonatal morbidity and mortality. Antibiotic sensitivity patterns vary geographically depending upon the prevalent local pathogens and common antibiotic used in neonatal unit [8]. The widespread emergence of antibiotic resistance to commonly used antibiotics has become great challenge in the management of neonatal septicemia. The varying microbiological pattern of septicemia in neonates warrants the need for an ongoing review of the causative organisms and their antimicrobial susceptibility pattern.

Hence, the present study aimed to know the bacteriological profile of early and late onset neonatal septicemia along with their antibiotic susceptibility patterns in Chitwan Medical College Teaching Hospital (CMCTH), Bharatpur, Nepal.

## 2. Methods

This prospective study was carried out in CMCTH, a 600-bed hospital located in Bharatpur, Chitwan district of Nepal from February to July 2017.

### 2.1. Study Population

The study consists of a total of 516 neonates (less than 28 days) with clinical manifestation of septicemia. Selection was based on the signs and symptoms such as fever, poor feeding, respiratory distress, cyanosis, cold clammy skin, tachycardia, seizures, hyperreflexia, jaundice, instability, etc.

### 2.2. Sampling Procedure and Processing

About 1-2 ml of blood was drawn aseptically before starting antimicrobial therapy and directly inoculated into Brain Heart Infusion broth (BHI) (HiMedia, India) in a ratio of blood:BHI of 1:5. The blood culture bottles were immediately sent to the microbiology laboratory and incubated at 37°C for 24 hrs and subcultured on MacConkey agar, blood agar, and chocolate agar (HiMedia, India) daily for 7 days. The inoculated MacConkey agar plates were incubated aerobically, whereas blood agar and chocolate agar plates were incubated in CO_2_ enriched humid atmosphere using candle jar, at 37°C for 24-48 hours. Blood culture bottles showing no growth on subculture done after incubation of 7 days were reported as negative. All the collected blood samples were processed for culture and isolation by standard microbiological methods [9].

### 2.3. Antibiotic Susceptibility Testing

The antimicrobial susceptibility testing was done by Kirby-Bauer disc diffusion method as recommended by Clinical Laboratory Standard Institute (CLSI) guidelines [10]. Antibiotic disks (HiMedia, India) used were ampicillin/sulbactam (10/10*μ*g), amikacin (30*μ*g), ceftriaxone (30*μ*g), cefotaxime (30*μ*g), cotrimoxazole (25*μ*g), clindamycin (2*μ*g), cefoxitin (30*μ*g), cefixime (5*μ*g), cloxacillin (5*μ*g), erythromycin (15*μ*g), gentamicin (10*μ*g), meropenem (10*μ*g), nalidixic acid (10*μ*g), ofloxacin (5*μ*g), piperacillin/tazobactam (100/10*μ*g), teicoplanin (30*μ*g), and vancomycin (30*μ*g).

For quality control of biochemical tests, purity plate was used [11]. Similarly, for quality control of antimicrobial susceptibility testing,* E. coli *ATCC 25922 and* S. aureus *ATCC 25923 were used.

### 2.4. Ethical Committee Approval

Ethical approval was obtained from the Chitwan Medical College-Institutional Review Committee before starting the study. Informed consent was obtained from parents of neonates before sample collection.

### 2.5. Statistical Analysis

The collected data were summarized, presented, and analyzed using the software SPSS version 20 (Chicago, USA).

## 3. Results

### 3.1. Prevalence of Neonatal Septicemia

Out of total 516 blood sample received from suspected neonates, significant bacterial growth occurred in 56 (10.8%) samples, contaminants were grown in 32 (6.2%) samples, and no bacterial growth occurred in 460 (82.9%) samples.

### 3.2. Sexwise Distribution of Neonatal Septicemia

Out of total 516 neonates, septicemia was confirmed in 56 (10.8%) neonates. Of these 56 neonates, 32 (57.1%) were inborn, while the other 24 (42.8%) were outborn, out of which 37 (66%) were males and 19 (33.9%) were females with predominant male to female ratio of 1.9:1. Prevalence of occurrence of EOS was much higher 35 (62.5%) neonates in comparison to LOS 21 (37.5%) neonates.

### 3.3. Distribution of Isolates from Blood Culture

Majority of neonatal septicemia were caused by gram-negative isolates 39 (69.6%) compared to gram-positive isolates 17 (30.3%). Neonatal septicemia was more commonly caused by gram-negative isolates among male 37 (66%) than in female 19 (33.9%). From 56 blood samples, 9 bacterial species were isolated.* Acinetobacter *species 18 (32.1%) was most commonly isolated organism followed by* S. aureus *11 (19.6%), CONS 6 (11.11%),* E. coli *5 (8.9%),* Enterobacter *species 5 (8.9%),* K. pneumonia*e 4 (7.1%),* Pseudomonas *species 3 (5.3%)*, Citrobacter *species 2 (3.5%), and* Salmonella *paratyphi A 2 (3.5%), respectively (Table 1).

### 3.4. Causative Organisms of EOS and LOS

The predominant isolate in EOS was* Acinetobacter *species 18 (32.14%) followed by* S. aureus *9 (16%),* E. coli *3 (5.3%),* Enterobacter *species 3 (5.3%),* Citrobacter *species 2 (3.5%),* Salmonella *paratyphi A 2 (3.5%),* K. pneumoniae *1 (1.7%),* Pseudomonas *species 1 (1.7%), and CONS 1 (1.7%). The predominant isolate in LOS was* S. aureus *11 (19.6%) followed by* Acinetobacter *species 5 (8.9%), CONS 5 (8.9%),* K. pneumoniae *3 (5.3%),* E. coli *2 (3.5%),* Enterobacter *species 2 (3.5%), and* Pseudomonas *species 2 (3.5%) (Table 2).

### 3.5. Antibiotic Profile of Gram-Positive and Gram-Negative Isolates Recovered

Among gram-positive isolates,* S. aureus *showed highest rate of susceptibility towards vancomycin, amikacin, teicoplanin, meropenem, cotrimoxazole, clindamycin, erythromycin, and ofloxacin. Similarly, CONS showed highest rate of susceptibility towards vancomycin, amikacin, teicoplanin, and piperacillin/tazobactam. Most of the gram-negative* Enterobacteriaceae *showed highest susceptibility towards amikacin, piperacillin/tazobactam, ampicillin/sulbactam, meropenem, ofloxacin, and gentamicin (Table 3).

## 4. Discussions

Neonatal septicemia is considered the leading cause of infant mortality and morbidity in the NICU. The frequency of infections in NICU varies from 6% to 25% in the United States and from 8% to 10% in Europe [12]. There has been a wide variation in the growth positivity in India; it has ranged from 16% to 54% [13]. In this study, blood culture positivity rate in neonatal septicemia cases is 10.8%; similar results were found by Mudzikati* et al. *2015 [14] (9.8%) and Ansari* et al*. 2015 [15] (12.6%). Lower incidence rate was reported in Nepal by Nepal* et al. *2013 (2.1%) [16] and Raha* et al *(8.9%) [17]. Much higher incidence rate was reported by Sarasam* et al. *2014 (36.4%) [18] and Al-Shamahy* et al*. 2012 (57%) [19], respectively. The variation in culture positivity rate of neonatal septicemia might be due to differences in sample size, prior antibiotic administration before sample collection, infection with anaerobes, viral or fungal pathogens, and effective control in spread of nosocomial infection.

In this study neonatal septicemia was more common in males 66% than in females 33.9% which correlates with the findings of previous studies which revealed that incidence of septicemia was higher in males ranging from 59% to 82% [20]. Probably, this might be due to more priority given to male babies for medical care in our society. The pathophysiology of neonatal sepsis has not been investigated and this could be further investigated in future which can give new insights in the management, diagnosis, and treatment of neonatal septicemia.

In our study early onset septicemia 62.5% was more common than late onset septicemia 37.5% which was also seen by Assudani* et al. *2017 [21] and Hafsa* et al. *2011 [22]. On contrary some report shows that late onset septicemia is more common than early onset septicemia, Muhammad* et al. *2010 [23]. The higher rate of EOS observed in our study may be due to early horizontal transmission of pathogens from NICU and delivery rooms or vertical transmission of these pathogens colonized in maternal genital tract after unhygienic obstetric practices. LOS is caused by postnatal acquisition of the pathogens, caused by the bacteria which thrive in the external environment of the hospital or home. A possible explanation for lower incidence of late onset septicemia could be better understanding in the importance of cleanliness, hygiene, and using aseptic techniques in a hospital setting by medical staffs.

The majority of isolates causing neonatal septicemia were gram-negative isolates 69.6%, similar to findings of Roy* et al*. 2002 [24] and investigators of the Delhi Neonatal Infection Study (DeNIS) Collaboration [25]. Likewise, preponderance of the gram-negative bacilli has been reported in other studies conducted in Nepal and Pakistan [26]. On contrast, other studies from abroad revealed gram-positive cocci including* S. aureus*, CONS, and group B streptococci as the predominant isolates [27].

*Acinetobacter *species 32.1% was most commonly isolated organism followed by* S. aureus *19.6%; similar findings have been reported by Mishra* et al. *1998 [28]. The causative agents of neonatal septicemia have changed over time and may vary from place to place. Shrestha* et al*. 2008 [29] isolated* E. coli *as the most predominant organism while Kumaravel* et al. *2016 [30] isolated* K. pneumoniae. *In contrast, Peterside* et al. *2018 in Nigeria and Sharma* et al. *2013 in India found* S. aureus*. Likewise, CONS predominance was reported by Mohamadi* et al*. 2018 [31]. This variation could be due to differences in study setting, study population, and adherence to hand hygiene practices.

We obtained* Acinetobacter *species and* S. aureus *as the predominant isolate in EOS which is consistent with previous findings made by Arora* et al. *2006 [32]. However, other studies reported* Klebsiella *species and* S. aureus *as most common cause of EOS [33]. Zakariya* et al. *2011 reported* Klebsiella pneumoniae *and CONS as the commonest isolate in EOS [34].* Acinetobacter *is a nosocomial pathogen and probably newborns are being infected by hospital pathogen or cross-contamination between patients or due to lapses in infection control practices in hospital which might be the reason for finding* Acinetobacter* as a predominant isolate in EOS.

In our study, amikacin was the most effective drug for both gram-positive and gram-negative isolates which correlates with findings of Muley* et al*. 2015 [35].* S. aureus *and CONS showed highest susceptibility towards vancomycin, amikacin, and teicoplanin. Vancomycin showed 100% sensitivity towards gram-positive isolates similar to the findings of Singh* et al. *2016 [36]. Vancomycin is still the drug of choice for* S. aureus*, but recently resistance to this drug has also been reported. A similar trend may also be expected in the developing world due to its lower cost and increased availability [37].

Other studies conducted both inside and outside Nepal showed a similar finding which reported that both gram-positive and gram-negative organisms showed high susceptibility to carbapenems [38]. Ofloxacin displayed high susceptibility towards gram-negative isolates in comparison to gram-positive isolates. In the present study, antibiotic resistance among the gram-positive and gram-negative bacteria was quite high to recommended drugs like ampicillin, cephalosporins, and aminoglycosides. The study of Nepal et al. 2013 and Ansari et al. 2015 of neonatal septicaemia, published from the same hospital, and Gyawali and Sanjana 2013 [39] from different hospital but the same location has also shown similar result of resistance trend. Higher trend of the resistance in these last 5 years may be primarily due to emergence of resistant strains as a result of indiscriminate and over use of antibiotics at private clinics and primary health care facilities from which neonates are referred to our center.

The resistance rate of cotrimoxazole reported by Nepal* et al*. in 2013 was 100% and Ansari* et al*. in 2015 from the same hospital was 66%; it is dramatically decreased to 18% in this study. This desirable decrement in resistant rate could be attributed to the less use of these antibiotics in clinical setting for neonates. Most of the gram-negative isolates showed highest susceptibility towards amikacin, piperacillin/tazobactam, meropenem, ofloxacin, and gentamicin. This finding is similar to another study done by Rao* et al. *2015 [40]. The main reason for variation in antibiotic susceptibility pattern might be due to differences in pattern of antibiotic used in different hospitals or due to emergence of antibiotic resistant strains as a result of indiscriminate use of antibiotics.

## 5. Conclusions

*Acinetobacter *species and* S. aureus *were the predominant cause of neonatal septicemia in our setup. High degree of antibiotic resistance was observed to commonly used antibiotics among both gram-positive and gram-negative isolates. Emerging antibiotic resistance is associated with significant neonatal mortality and morbidity. Amikacin was the most effective drug against the gram-positive and gram-negative bacteria. Therefore, it is mandatory to perform routine antimicrobial susceptibility surveillance and periodic review of hospital and national antibiotic policy to reduce the burden of antibiotic resistance. Further epidemiological and clinical studies are vital to curb the changes in microorganisms causing neonatal septicemia.

## Figures and Tables

**Table 1:** Distribution of organisms isolated from blood culture in neonatal septicemia.

**Organism isolated**	**Sex n (**%**)**		**Total no. of organism n (**%**)**
	**Male**	**Female**	
**Gram-negative organisms (n=39)**			
**Enterobacteriaceae**			
*Escherichia coli*	4 (7.1)	1 (1.7)	5 (8.9)
*Enterobacter* species	2 (3.5)	3 (5.3)	5 (8.9)
*Klebsiella pneumoniae*	3 (5.3)	1 (1.7)	4 (7.1)
*Citrobacter *species	1 (1.7)	1 (1.7)	2 (3.5)
*Salmonella *paratyphi A	0	2 (3.5)	2 (3.5)
**Others**			
*Acinetobacter* species	14 (25.0)	4 (7.1)	18 (32.1)
*Pseudomonas* species	2 (3.5)	1 (1.7)	3 (5.3)
**Gram-positive organisms (n=17)**			
*Staphylococcus aureus*	9 (16.0)	2 (3.5)	11 (19.6)
CONS	2 (3.5)	4 (7.1)	6 (11.1)
**Total no. of organisms**	**37 **(66.0)	**19 **(33.9)	**56**(100)

CONS*: *coagulase negative* staphylococci*. Figure in parenthesis indicates percentage.

**Table 2:** Causative organisms of EOS and LOS.

**Organism isolated**	**Onset n(**%**)**		**Total no. of organism n (**%**)**
	**EOS**	**LOS**	
**Gram-negative organisms**			
**Enterobacteriaceae**			
*Escherichia coli*	3 (5.3)	2 (3.5)	5 (8.9)
*Enterobacter* species	3 (5.3)	2 (3.5)	5 (8.9)
*Klebsiella pneumoniae*	1 (1.7)	3 (5.3)	4 (7.1)
*Citrobacter *species	2 (3.5)	0	2 (3.5)
*Salmonella *paratyphi A	2 (3.5)	0	2 (3.5)
**Others**			
*Acinetobacter* species	13 (23.2)	5 (8.9)	18 (32.1)
*Pseudomonas* species	1 (1.7)	2 (3.5)	3 (5.3)
**Gram-positive organisms**			
*Staphylococcus aureus*	9 (16.0)	11 (19.6)	11 (19.6)
CONS	1 (1.7)	5 (8.9)	6 (11.1)
**Total no. of organisms**	**35 (62.5)**	**21 (37.5)**	**56**(100)

LOS: late onset septicemia, EOS: early onset septicemia, and CONS: coagulase negative *staphylococci*. Figure in parenthesis indicates percentage.

**Table 3:** Antibiotic sensitivity profile of gram-positive and gram-negative isolates.

**Antibiotics**	**Gram positive**		**Gram negative**		
	***S. aureus***	**CONS**	***Enterobacteriaceae***	***Acinetobacter *spp.**	***Pseudomonas *spp.**
A/s	18.1	NT	0	100	100
AK	90.9	100	16.6	94.4	100
CTR	0	83.3	22.2	11.1	100
CTX	18.1	83.3	16.6	11.1	NT
COT	81.8	33.3	22.2	72.2	0
CD	72.7	0	NT	NT	NT
CX	36.3	66.6	NT	NT	NT
CFM	18.1	50.0	27.7	5.5	100
COX	63.6	0	NT	NT	NT
E	72.7	0	NT	NT	NT
GEN	NT	0	100	88.8	NT
MRP	81.8	66.6	100	94.4	100
NA	9.0	NT	22.2	66.6	NT
OF	54.5	66.6	100	88.8	100
PIT	18.1	100	27.7	100	100
TEI	90.9	100	NT	NT	NT
VA	100	100	NT	NT	NT

Figures depict percentage, CONS: *Coagulase negative staphylococci*, NT: not tested, A/S: ampicillin/sulbactam, AK: amikacin, CTR: ceftriaxone, CTX: cefotaxime, COT: cotrimoxazole, CD: clindamycin, CX: cefoxitin, CFM: cefixime, COX: cloxacillin, E: erythromycin, GEN: gentamicin, MRP: meropenem, NA: nalidixic acid, OF: ofloxacin, PIT: piperacillin/tazobactam, TEI: teicoplanin, and VA: vancomycin.

## Data Availability

The data used to support the findings of this study are available from the corresponding author upon request.

## Abbreviations

AST: Antimicrobial susceptibility testing
ATCC: American Type Culture Collection
BHI: Brain Heart Infusion broth
CLSI: Clinical and Laboratory Standards Institute
CMCTH: Chitwan Medical College Teaching Hospital
CONS: Coagulase-negative* Staphylococci*
EOS: Early onset neonatal septicemia
LOS: Late onset neonatal septicemia
NICU: Neonatal Intensive Care Unit
OPD: Outpatient Department.
